# Short and Long Term Measurements in Assessment of FRP Composite Footbridge Behavior

**DOI:** 10.3390/ma13030525

**Published:** 2020-01-22

**Authors:** Mikołaj Miśkiewicz, Łukasz Pyrzowski, Bartosz Sobczyk

**Affiliations:** Faculty of Civil and Environmental Engineering, Department of Mechanics of Materials and Structures, Gdańsk University of Technology, G. Narutowicza 11/12, 80-233 Gdańsk, Poland; mikolaj.miskiewicz@pg.edu.pl (M.M.); bartosz.sobczyk@pg.edu.pl (B.S.)

**Keywords:** composite materials, structural health monitoring, strain measurement, displacement measurement, vibration measurement, sensor systems, sensors

## Abstract

The paper presents application of different sensors for the purpose of short and long term measurements, as well as a structural health monitoring (SHM) system to assess the behavior of a novel fiber reinforced plastics (FRP) composite footbridge. The aim is to present a thorough and concise description of these sensors networks and results gathered with their aid during in situ measurement of strains, displacements, and vibrations, as only a few works are available in this field. The bridge geometry, material solutions, and properties are described at first. Then the measurement devices composing the system and subsystems of sensors are elaborated on. Subsequently, the bridge research program is described and the results are shown and discussed. Finally, it is concluded that the use of selected sensors is helpful in assessment of the behavior of the novel structure, and moreover in validation of its numerical models. The collected data confirmed many assumptions made during the bridge design process and allowed us to accept it for exploitation.

## 1. Introduction

The advantages of composites, such as: relatively high strength and stiffness, low volume weight, good fatigue resistance, high material damping, and good environmental resistance make them attractive for different applications, including bridge and in particular footbridge industry and design (refer to references [1,2,3,4,5,6,7,8,9,10]). Therefore, the adoption of this relatively new material in this branch of engineering has already been started and developed. However, application of laminated composites as the main structural material, for instance fiber reinforced plastics (FRP) in civil engineering, is still not common.

All built engineering structures are subjected to different influences and loading conditions during their lifetime. Sometimes they are so unfavorable that damage or failures of objects are observed. From this reason, a lot of research is currently being done in the field of non-destructive damage detection methods and structural health monitoring (SHM). As the application of FRP as a structural solution in engineering structures is not very popular compared to steel, concrete, or wood, appropriate evaluation of FRP response and behavior is very important. The recent papers in this field are briefly discussed below.

Nowadays, a lot of research is being done to develop and improve measuring techniques that enable non-destructive testing and SHM of FRPs. However, in the majority of relevant papers, the results of experimental laboratory tests done on small specimens or coupons and numerical simulations are presented. These results are easily available in many journals and selected recent examples of this research can be found in references [11,12,13,14,15,16,17,18]. Similar papers dealing with problems on a slightly bigger scale (aircraft panels) are available in references [19,20].

Articles describing the use of network of sensors to gather information about short-term or long-term response of FRP bridges are rather rare. It has to be emphasized that it is very important to learn about applicability and accuracy of measurements carried out on real scale structures and not to limit research to laboratory coupon tests only. What is more, FRP laminated composite is a hi-tech material with a relatively short history of use. In the case of application of hi-tech materials or new technologies, careful attention should be paid during the design process and in the description of its response to assure and provide a guarantee that the structure is safe for the users. Therefore, papers dealing with these kinds of problems are very desirable and their found examples are briefly discussed. Short-term static and dynamics tests of composite footbridges have been recently presented in references [21,22]. Full SHM systems of composite bridges are shown in references [23,24,25]. Monitoring of a bridge deck is described in reference [26], while a review paper on FRP composite bridges monitoring is available in reference [27]. As shown above, only a few papers are currently available in this field. This indicates that research in this area is very desirable.

Therefore, this article aims to present non-destructive testing, strain and displacement measurements, and SHM system results, obtained with the aid of different systems of sensors, that were obtained to establish the short and long term performance of an innovative, composite sandwich (FRP-foam core-FRP) bridge. Applicability and accuracy of sensors is discussed as well. It is possible, based on the presented results, to assess the bridge structural capabilities and thus decide whether all the initial design assumptions are positively verified in in-situ tests. This research also points out that in view of the experimental measurements, the presented structural solution in conjunction with the chosen production technology is approved for possible application and therefore is available for all that are willing to use it.

## 2. Description of the Analyzed Bridge—Structural Solution, Materials, and Modeling

**Figure 1:**
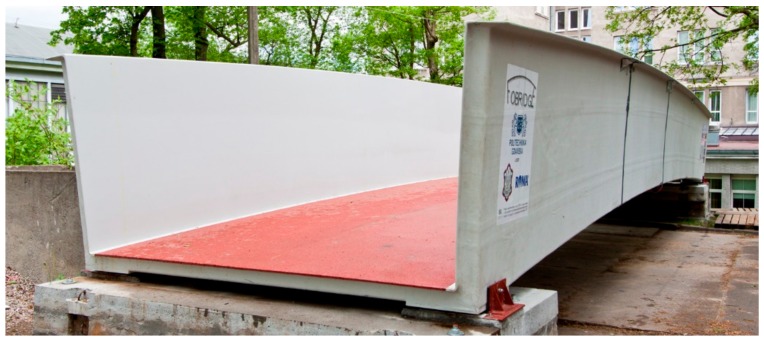
Composite footbridge designed within the FOBRIDGE project.

In 2015, the consortium associating Gdańsk University of Technology (GUT), Military University of Technology in Warsaw and ROMA Co. Ltd. (Grabowiec, Poland) constructed a single element, sandwich composite footbridge with a span of 14 m long using vacuum infusion technology (Figure 1). This is the final product of the FOBRIDGE project, which aims to design pedestrian and cyclist bridges made of composite materials for use over dual carriageways and motorways. The footbridges were designed from scratch. The whole process resulting in bridge production included development of a preliminary concept, material selections, identification of material properties, numerical simulations, strength calculations, and serviceability analyses. The structure is innovative aforementioned for these reasons. According to the author knowledge, it has been the first structure in the world to be classified as a consistent, single element structural girder, without any connectors or fasteners, made of sandwich shells, entirely produced in a single cycle vacuum infusion process. The application of non-classical materials caused problems, as no guidelines nor recommendations were available in existing design codes during project realization. This is why the project was expanded to include additional material parameters identification, validation analyses at different complexity levels, and experimental tests.

**Figure 2:**
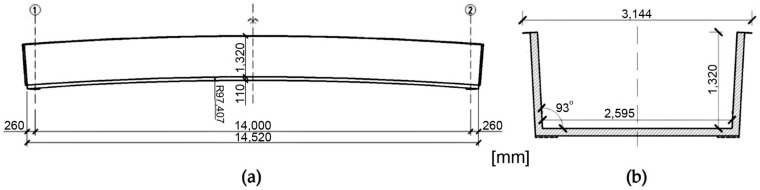
Footbridge views: (**a**) Side view; (**b**) Cross section.

The considered footbridge is a composite, U-shape, simply supported, shell-type sandwich structure, the dimensions of which are shown in Figure 2. The total weight of the footbridge superstructure is 3200 kg. It is completely made of hi-tech composites, without any conventional elements. The footbridge was designed to carry a uniformly distributed 5 kN/m^2^ service load.

The bridge span was built from sandwich panels with laminated polymer composite skins and a Polyethylene terephthalate (PET) foam core, 100 mm thick with a density of 100 kg/m^3^. The skins reinforcement, immersed in flame retardant vinylester resin matrix, was made from stitched and balanced [0/90] and [+45/–45] E-glass fabrics. Each lamina (0.663 mm thick) of the FRP skin had averaged elastic and strength properties in T = 20 °C, as presented in Table 1. These properties were established in the laboratory of the Military University of Technology (refer for example to reference [28]). The skins were additionally extended and folded to form pure laminated composite lips, in the top part of the bridge cross section (refer to Figure 2). The support zones were strengthened by precast glass fiber reinforced plastics (GFRP) stiffening elements to carry reaction forces. The load-bearing structural layers were additionally covered with an external coating provide protection from negative environmental and exploitation effects and to improve the bridge’s overall look. These extra layers were: topcoat, gelcoat and anti-skidding layer. Owing to the above, fourteen independent lamination schemes in total can be distinguished within the structure.

**Figure 3:**
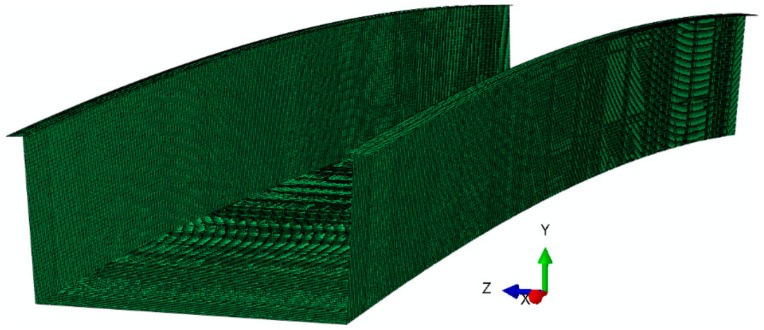
Visualization of the footbridge numerical model.

Many finite element method (FEM) computational models with different levels of complexity were created in order to estimate the bridge response under different conditions. They were used to carry on various studies intending to design the footbridge and establish a load tests program, including using a ballast selection or location of network of sensors. All the aforementioned studies were however preceded by complex validation analyses of laminate coupons, sandwich beams and 3 m long full-scale footbridge segment (see references [29,30]). In this paper some FEM estimations of strains and displacements under loads applied during short-term static load test are compared with the in-situ values in order to evaluate accuracy of measurements and modeling. A pure shell equivalent single layer (ESL) (refer to reference [31]) FEM model was used for this purpose (refer to Figure 3), which was motivated by the fact that in this case, only a global response of the bridge needed to be determined and consideration of local effects and influences could be avoided. The precision of numerical results was sufficient and relatively low computational effort was required. It is worth mentioning that the ESL was also found to be used in more sophisticated approaches of shell structures analysis [32].

Nevertheless, details of the shell model are not described in this paper, because the article is focused mainly on non-destructive testing, application of SHM, and results measured by the used sensor networks. A full description of the shell model, as well as considerations on choice of the appropriate approach to model such a bridge are available in references [33,34] and therefore we do not repeat them here.

The composite footbridge was manufactured by vacuum infusion by ROMA Co. Ltd. and assembled at the Gdańsk University of Technology campus, where all the in situ tests were conducted. After the scientific research was finished, the Project Leader (GUT) made efforts to use the footbridge for real pedestrian and bicycle traffic. Finally, it was assembled at a site near Pruszcz Gdański city over the Radunia River (Poland). After successful final acceptance and inspection (each bridge with a span length equal or greater than 20 m and prototype objects needs to undergo final acceptance and inspection in Poland) it now serves the local community.

## 3. Measurement Devices

### 3.1. General Information

To determine short and long term performance of the innovative bridge, some sensor networks were created, enabling structural health monitoring (SHM) system, strain, displacement and vibration measurements. The sensors used in the measurement systems can be divided into two groups. The first one creates the SHM system. It was designed to identify potential permanent strains or deformations, as well as changes of structure global stiffness (via changes of its natural frequencies). It was possible, with the help of the system, to observe effects resulting from e.g., unexpected rheological processes or the failure of structural components during long-term static tests, as well as periods between static tests, when the footbridge was occasionally used by pedestrians.

The system was composed of the following components:

- Fiber Bragg Gratings (FBG) optic fiber sensors to measure strain and temperature with sockets to connect a recorder and a portable data registration system (four measurement points)
- Vibrating Wire Strain Gauges (VWSG) to measure strain and temperature with a recorder (24 measurement points)
- Accelerometers and gyroscopes with connecting sockets and workstation to record the measured acceleration and speed of signals (36 measurement points)
- Geodetic benchmarks to perform displacement monitoring (14 measurement points)
- Video surveillance.

The second group contained sensors that were used during short-term load tests. It was designed to gather more precise information about structure behavior, compared with the first one. It included all sensors from the first group (FBG, VWSG, accelerometers, and gyroscopes) and the following additional components:

- Electrical resistance strain gauges (SG) (21 measurement points)
- Inductive displacement sensors (nine measurement points)
- Geodetic precision leveling (48 measurement points)
- Bearing deformation dial indicators (four measurement points)
- Abutment settlement geodetic monitoring (four measurement points)
- Terrestrial laser scanning similar to that used in references [35,36]
- Algorithms of post-processing numerical analyses based on big geospatial datasets [37,38].

All the devices grouped above were divided into three independent subsystems enabling measurements of strains, displacements, and vibrations. They are described in detail below.

### 3.2. Strain Measurement Subsystem

A strain measurement subsystem was equipped with three types of gauges. The first one is a FBG optic fiber sensor, which was planned to be used during the mass production of the composite footbridge after confirmation of its performance. These sensors may be installed inside the structure. The second one contains VWSG sensors, enabling interval registration (predetermined time) of strain changes. The last type is a SG sensor, which is dedicated to short-term load tests.

#### 3.2.1. Optic Fiber Sensors FBG

**Figure 4:**
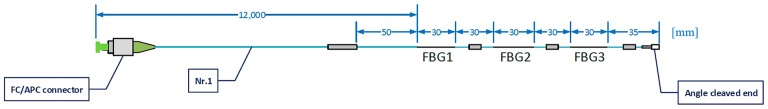
FBG optic fiber sensor—a scheme.

**Figure 5:**
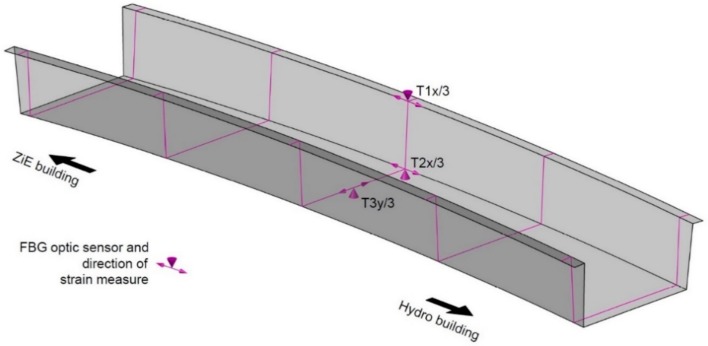
Location of FBG optic fiber sensors.

The fiber optic measurement system is based on two kinds of sensors. It includes three Bragg gratings to measure strain and one for temperature compensation. They form a one wire chain, ending in the SC/APC connector, see Figure 4. The following parameters characterize the Sylex (Bratislava, Slovakia) strain gauges used in measurements: 8 mm measuring length, 0.85 με resolution, 1.7 με accuracy, range of measure up to 5000 με. The temperature gauge parameters are: 0.1 °C resolution, 0.2 °C accuracy, in a measuring range from −20 °C to + 150 °C. The single strain gauge length is 30 mm, it was fixed to the structure by the cyano-acrylate dedicated glue. The measurements were carried out using the Sylex SCN-80 S-line Scan 800 interrogator and a computer with dedicated ILLumiSense Strain software. The locations of the sensors are shown in Figure 5, they are all placed in one cross-section at the mid-span.

It is assumed that in future development of the project, FBG sensors will be installed inside the footbridge structure during the manufacturing process. For now, the idea has been temporarily abandoned. This was because the carried out preliminary tests revealed difficulties. It was difficult to maintain the technological regimes and avoid mechanical damage of sensors. However, this problem is planned to be resolved in future research.

#### 3.2.2. Vibrating Wire Strain Gauges VWSG

**Figure 6:**
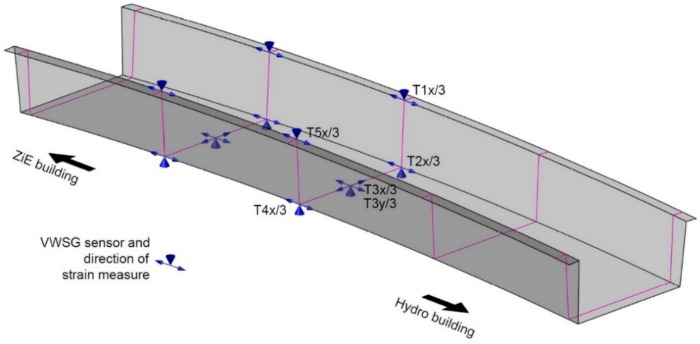
Location of VWSG sensors.

**Figure 7:**
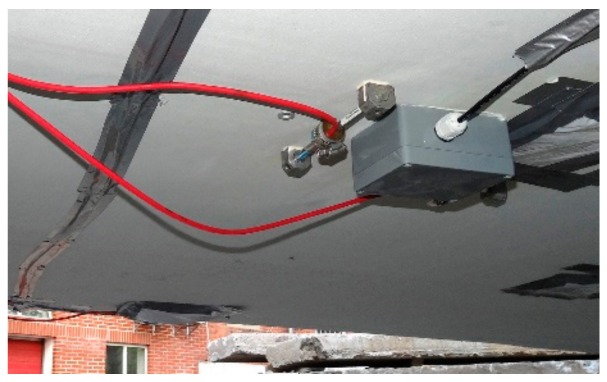
VWSG sensor fixed to the bridge.

The VWSG measurement system is based on twelve GEOKON strain and temperature model 4000 gauges. The strain is measured on the basis of changes in the natural frequency of a string attached to anchor blocks inside a device. The system is characterized by a very good stability during long-term monitoring. The sensors are connected to the 16-channel recorder, which initiates the measurement within a given time interval. The parameters of the sensors are: measuring length of 150 mm, measuring range up to 3000 µε, resolution of 1 µε, accuracy <0.5% FS, temperature range −20 °C to + 80 °C, simultaneous measurement of temperature and strain. The locations of the VWSG are shown in Figure 6. The sensors are placed in two cross-sections, at quarter and mid-span. One of the sensors, being fixed to the bridge deck is presented in Figure 7.

#### 3.2.3. Electrical Resistance Strain Gauges SG

**Figure 8:**
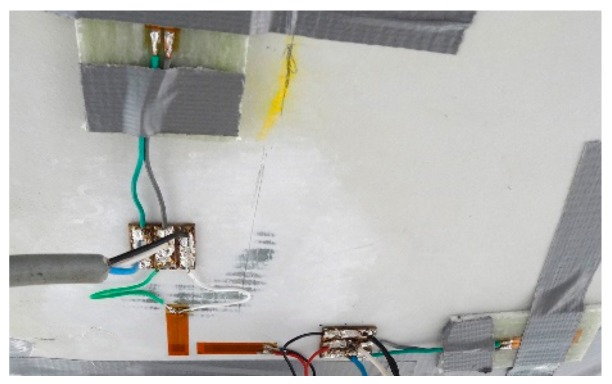
SG sensors mounted to the structure.

**Figure 9:**
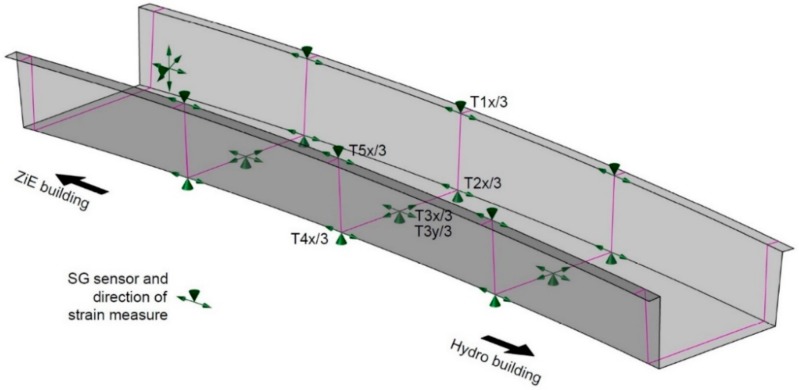
Location of SG sensors.

The measurement system is based on the electrical resistance strain gauges. The Hottinger Baldwin Messtechnik (HBM) (Darmstadt, Germany) foil gauges are used. They have the following properties: active grid length of 20 mm, 120 Ω nominal resistance and 50,000 microstrain range. Temperature is compensated in each measurement point using individual sensors, as is shown in Figure 8. The measurements were carried out using the HBM QuantumX MX1615B strain gauge bridge amplifier and a computer with dedicated the CatmanEasy/AP software. The locations of the sensors are shown in Figure 9, they are placed at the quarter spans and at the mid-span and over the supporting area (a rosette).

### 3.3. Displacement Measurement Subsystem

**Figure 10:**
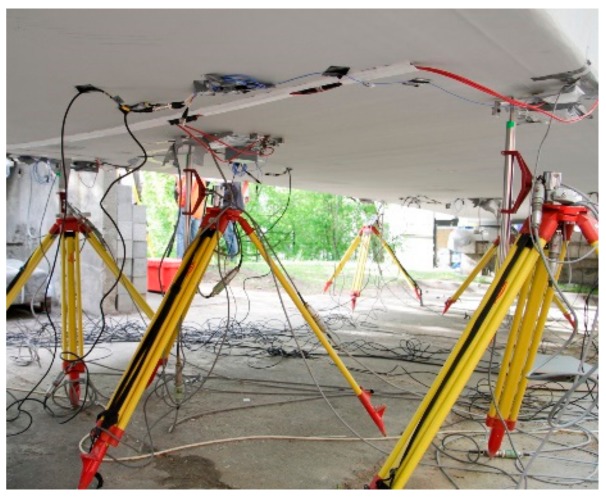
Inductive transducers used during measurements.

**Figure 11:**
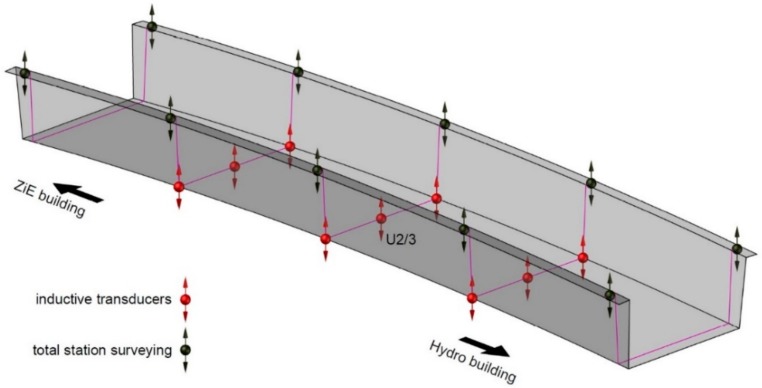
Location of displacement measurement points: inductive transducers and total station surveying.

The displacement measurement subsystem includes inductive transducers (see Figure 10) and precision leveling. The nine 100 mm range HBM inductive transducers connected to the HBM MX840B universal amplifiers are located as shown in Figure 11. The precision leveling measurement by Leica Geosystems (Heerbrugg, Switzerland) Nova TM50 Robotic Total Station was achieved with an accuracy of 0.1 mm in the locations presented in Figure 11 as well.

### 3.4. Vibration Measurement Subsystem

**Figure 12:**
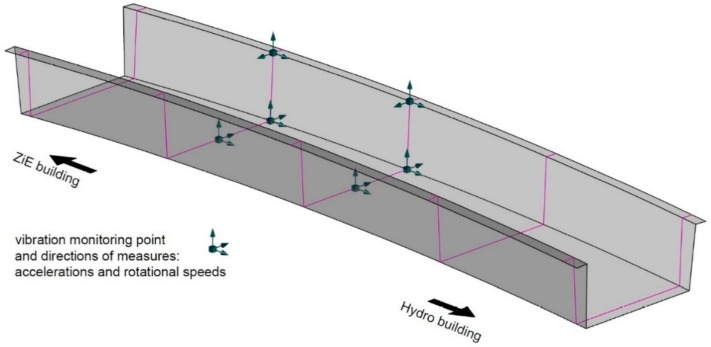
Location of vibration monitoring sensors.

The vibration measurement subsystem was based on a six-channels prototype sensors. They comprise of three integrated orthogonal channels to measure accelerations and three orthogonal channels to measure rotational speed. The accelerometers output range is ±2 g, the default frequency measure capabilities range from 0 Hz to 40 Hz (possible to 200 Hz), and the error of non-linearity is 0.5%. The gyroscopes range is ± 250 °/s, the default and possible frequency range is the same as above and the error of non-linearity is 0.2%. The data is sent to a workstation the via internet. This allows for previewing results in real time and data archiving. It also provides a possibility of measurement signal post processing at any time. The sensors are designed to work in temperatures from −30 °C to + 85 °C and have a degree of protection of IP67. The system, in total, contains six sensors, six-channels each, giving eighteen measurement points. Their locations are shown in Figure 12.

## 4. Research Program

### 4.1. General Information

The footbridge located at the Gdańsk University of Technology campus had been subjected to various tests for one year (May 2015–May 2016). A complex program was planned in order to gather maximum information about the structure behavior: statics, dynamics, creep, and thermal deformation. The diagnostic approach was based on both standard and new measurement procedures. Standard procedures involved the load test typical for bridges made of traditional materials (concrete, steel, wood). The use of new types of tests was required to confirm, that the structure will meet the design requirements throughout its intended life cycle. Thus, three different measurement periods may be distinguished:

- Short-term load tests
- Long-term load test
- Ambient (environmental influence) testing.

### 4.2. Short-Term Load Tests

According to Polish law, all bridges with a span length greater than or equal to 20 m and prototype objects should undergo final acceptance tests. During the tests, the correctness of the structure behavior in relation to design assumptions is verified. It is needed to load the bridge in such a way that the resulting effects, such as internal forces, displacements, and stresses are large enough. Namely the ratio between the measured and designed values should be greater than 75% and not exceed 100% [39,40,41,42,43,44,45,46,47,48].

This research was particularly important for the analyzed structure. First of all, because at the stage of the design and footbridge final tests, no guidelines or standards were available in this field. During the design phase, all conclusions about the behavior of the considered footbridge were based on FEM simulations, identification [30], and validation tests [29].

The main aim of performing short-term load tests was to verify load carrying abilities of the analyzed footbridge. Additionally, the obtained results allowed to finish the validation process of the numerical model, which was tested earlier on smaller structures: coupons, beams, 3 m footbridge segment [29,30].

Two tests, denoted as U1 and U2, were realized in May 2015 in order to obtain at the mid-span of the superstructure 75% to 100% of the designed maximum bending moment value, resulting from the characteristic live load. The U1 test was also repeated in August and November 2015. Moreover, third test denoted as U3 was performed in September 2015, aiming to reach the designed limits of the superstructure, corresponding to the ultimate limit state. During the U3 test, the internal forces in the structure equaled approximately 120% of the ones obtained under standard live loads. The first two tests were supposed to confirm that the bridge response is elastic under different and repeated loading conditions in various periods of time and atmospheric conditions. The third test was launched because, in the other two, a stiffness safety margin of the bridge was observed. The FEM estimations of bridge response were generally lower than the measured values. Therefore, the increase of load would have not resulted in any damage or failure. What is more, its implementation as part of the research project was a unique possibility for reliable verification of the actual safety factors used in the design (γ_f_ = 1.5). The research team was aware of the fact that in future the bridge owner would never agree to test such a possibility of a significant increase in bridge strain. It was the only opportunity to do this research.

**Figure 13:**
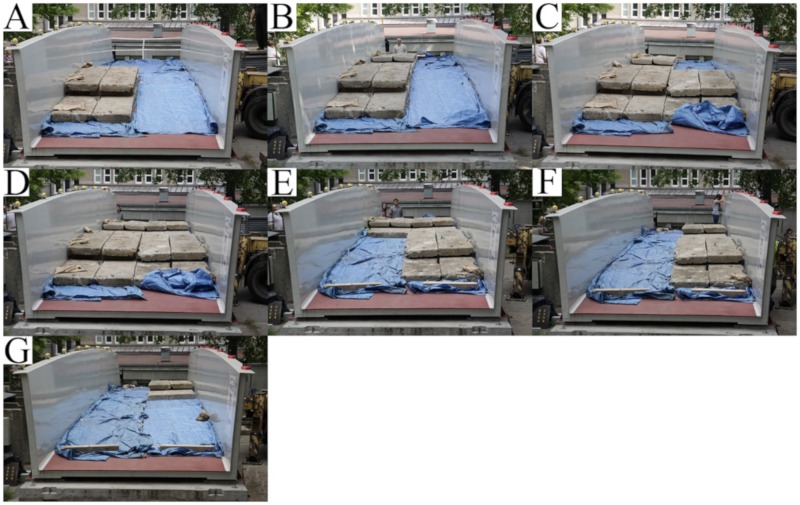
Loading sequence during the U1 test (from **A** to **G**).

**Figure 14:**
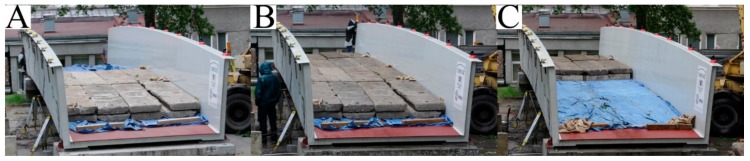
Loading sequence during the U3 test (from **A** to **C**).

In all static cases, the ballast was applied using concrete slabs. The total weights of the slabs during the tests were—U1: 141.2 kN, U2: 150.0 kN, and U3: 201.7 kN. The load was always put on the deck in subsequent steps. During the U1 test, the concrete slabs were placed in seven steps from A to G (each quarter of the deck) were loaded separately, see Figure 13. During U2 and U3 tests in three steps from A to C, each half of the deck loaded separately, see Figure 14. The ballast was laid on the structure in stages, asymmetrically, with respect to the longitudinal and transverse axes of the footbridge. The approach, in which so many different types of measuring points are used, was designed to identify possible irregularities in the behavior of the footbridge parts.

### 4.3. Long-Term Load Tests

Long-term studies on full-size bridge structures, regardless of the structural solution or used materials, are unique. Moreover, it is practically impossible to carry out such tests for bridges which are in operation. For this reason, all long-term studies carried out on real-size objects are valuable for assessing the response of any type of structure. In the case of the presented pedestrian bridge superstructure, it was particularly important due to the use of a modern composite material and a sandwich type solution. The idea of conducting a long-term load test came up especially because of a modern materials application. It was very important to verify the sensitivity of the material to rheological processes and to check whether after removing the ballast, the bridge response is still purely elastic. The footbridge was loaded in the same configuration as during U2 test, however the full ballast (150 kN), refer to Figure 13D, was left on the deck for a relatively long period of time, which was August–November 2015. It should also be noted that the application of the long-term load scheme allowed for determining whether the footbridge span, subjected to several changes of atmospheric conditions, was still operating in the elastic range.

### 4.4. Ambient Testing

The designed life-time of bridges is usually 100 years. During this period, they are subjected to different environmental conditions. In addition, as indicated in reference [28], there is a direct relationship between temperature changes and the modulus of elasticity of a composite material. Therefore, an advanced measuring system was used to assess the influence of atmospheric effects on the behavior of the full-size pedestrian bridge span. Its task was to collect information about the behavior of the structure over a relatively long period of time. The obtained data was useful for evaluating the footbridge operation over the target use period.

The footbridge was continuously monitored by the SHM system during periods when there was no static load on the footbridge. Temperature changes and wind were the main environmental influences acting on the footbridge. In that time, the structure was also occasionally loaded by crossing pedestrians. The footbridge was not exposed to public traffic due to the fact that the project was still in the execution phase. That is why the presentation of vibration measurement data from the entire measurement period is pointless.

The purpose of temperature measurements was to monitor its changes and its direct impact on the values of composite strains. Daily temperature changes and season-related effects were expected to be observed. Thus, monitoring was carried out throughout an entire year. The registered response of the structure is unique and allows us in subsequent stages of the work to formulate recommendations for composite spans made of a sandwich material regarding their appropriate design, including the effects of temperature.

## 5. Results and Discussion

### 5.1. Short-Term Load Tests

**Figure 15:**
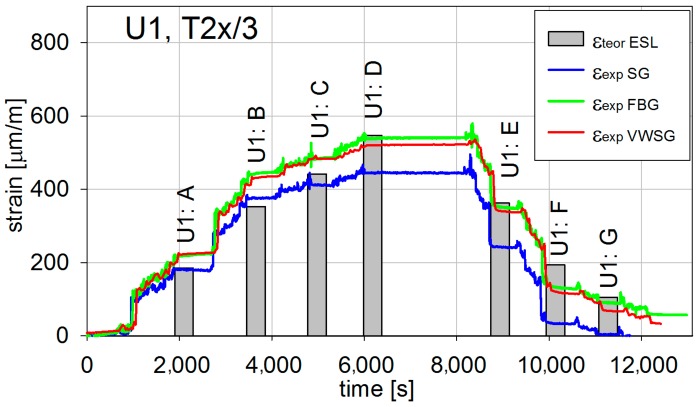
Longitudinal strains in T2x/3 for the U1 test.

**Figure 16:**
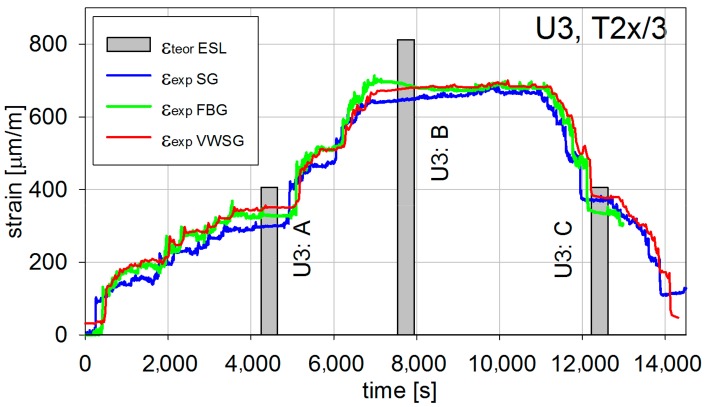
Longitudinal strains in T2x/3 for the U3 test.

During the short-term load tests, all three systems of strain measurement sensors were used. The exemplary results, being the extreme values, shown in Figure 15 and Figure 16, present registered strains (temperature compensation had been made during the measure) correspondingly during U1 and U3 tests in T2x/3 measurement point (refer to Figure 5, Figure 6, and Figure 9). The experimental results ε_exp_ are compared with the numerical ones ε_teor_ ESL. The results in the remaining measurement points reveal similar character and result precision, and hence are not depicted.

All strains measured with three different techniques are quite comparable. Therefore, it can be concluded that the in situ strain values are correct. The differences observed between them are caused mainly by slightly different locations of sensors. It is not possible to locate all of them precisely at the same point of the bridge. Additionally, the measurements can be also influenced by the sensor characteristics and their measuring precision. The numerical values are higher or close to the in situ ones. This shows that the structure is in general stiffer than it was designed to be and therefore enables safe exploitation.

**Figure 17:**
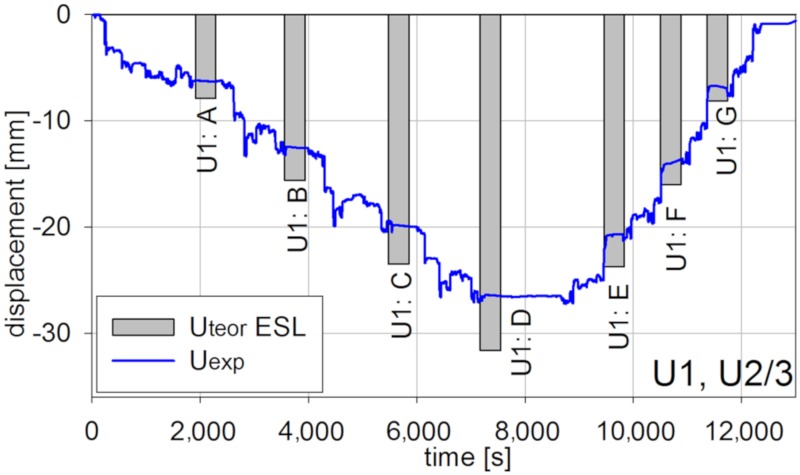
Vertical displacements in U2/3 for the U1 test.

**Figure 18:**
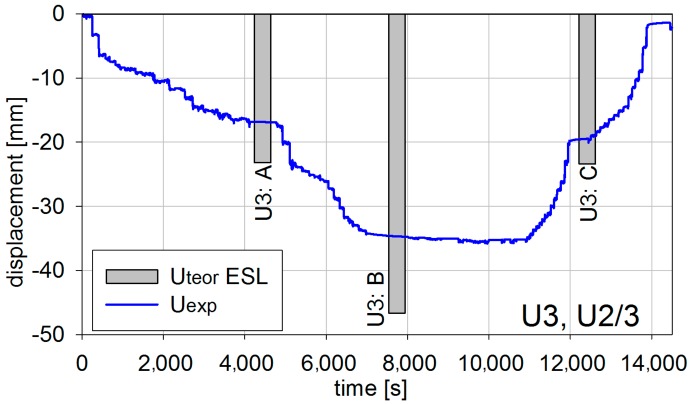
Vertical displacements in U2/3 for the U3 test.

Also, displacements were measured during the short-term test. The example results from inductive sensors are shown in Figure 17 and Figure 18 correspondingly for U1 and U3 tests in U2/3 points, for which extreme values were reported (refer to Figure 11).

The displacement inductive sensors measurements were checked using geodetic precision leveling and thus assessed as precise. Therefore, they can be used to assess the behavior of the innovative bridge. Similarly as in the case of a strain measure, the in situ measure revealed a stiffness margin for the safety of the bridge.

### 5.2. Long-Term Load Tests

**Figure 19:**
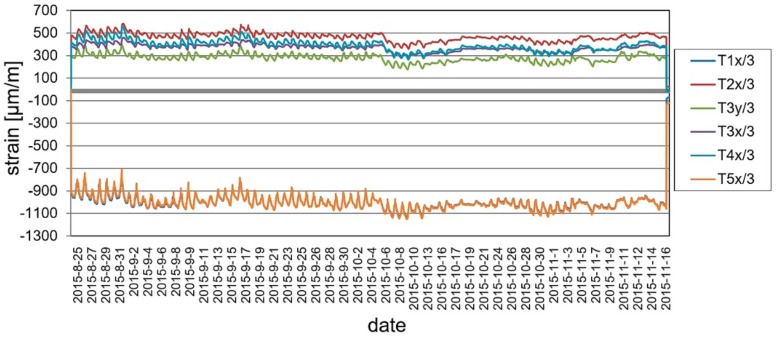
Strain monitoring using VWSG sensors during the long-term load test.

**Figure 20:**
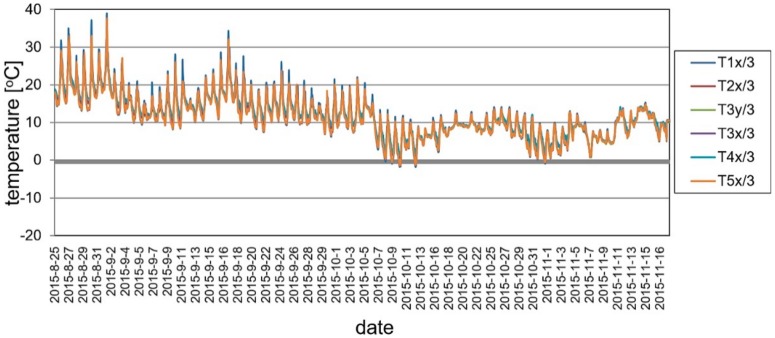
Temperature changes during the long-term load test.

**Figure 21:**
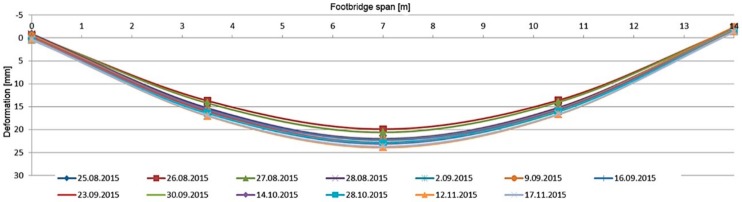
Displacements in U4 measurement points, footbridge with ballast.

**Figure 22:**
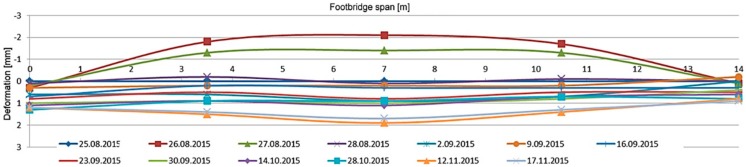
Normalized displacements in U4 points, footbridge with ballast.

During the long-term load tests (U2 ballast had been left on the deck) the measurement system included VWSG sensors and vibration monitoring, as well as periodic precision leveling. The changes of the mid-span strains in time are shown in Figure 19, while the change of temperature in the same domain of time as presented for strains is shown in Figure 20. The established time intervals measurements in characteristic points of the structure by precision leveling is presented in Figure 21. For more detailed verification, regardless of whether the weight of the ballast caused rheological changes in displacements, the obtained results were normalized with respect to the deflection at start of the test. The result is shown in Figure 22.

As was predicted, only a small strain and displacement deviations are observed because of the diurnal and seasonal changes of temperature. The performance of the bridge did not change because of rheological effects and inelastic strains or permanent displacements did not occur.

### 5.3. Ambient Testing

**Figure 23:**
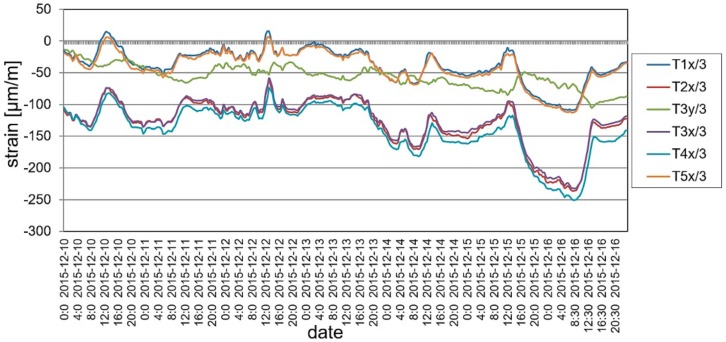
Strain monitoring using VWSG sensors during a one week period.

**Figure 24:**
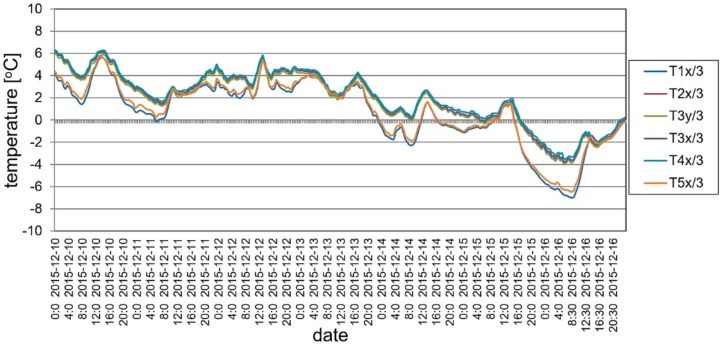
Temperature changes during a one week period.

**Figure 25:**
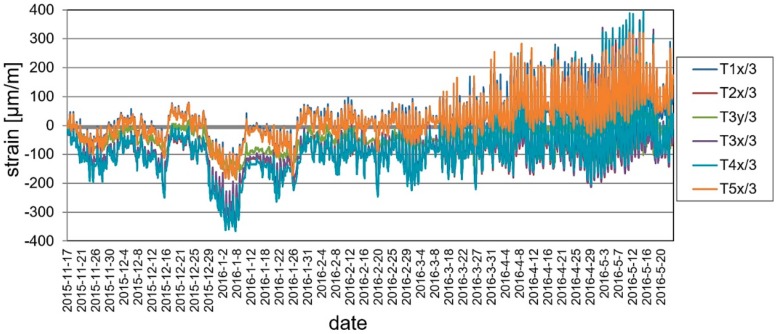
Strain monitoring using VWSG sensors during a six month monitoring period.

**Figure 26:**
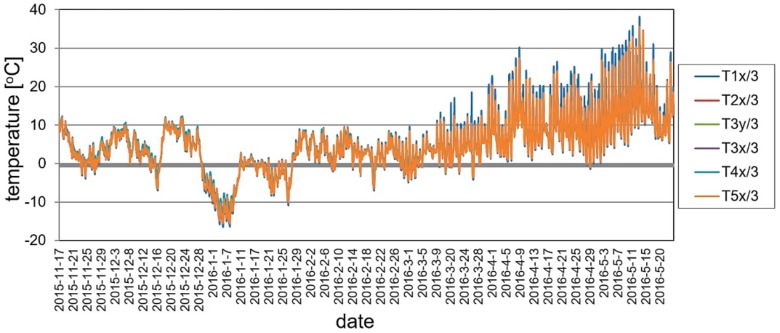
Temperature changes during a six month monitoring period.

The structure was still continuously monitored by the SHM system when there were no static loads on the footbridge, between May 2015 and May 2016. In order to maintain readability of the recorded data, the presentation of results was limited to the periods of November 2015 and May 2016. The changes of the mid-span strains in time for ambient testing during a sample week period is shown in Figure 23. The temperature change in the same domain of time as presented for strains is shown in Figure 24. According to the surveillance observations and the gathered data the temperature changes and wind were the main influences affecting the footbridge. It can be observed in Figure 25 and Figure 26, which are strains and temperature time histories throughout the six month monitoring period, that because of these effects, strains range from about −400 to 400 microstrains. Nevertheless, higher values were calculated at the stage of bridge designing. Therefore, the SHM strain measurement confirms the safe design and behavior of the bridge for environmental influences. The graphs clearly show the impact of both daily temperature changes and the ones associated with the change of seasons. The research confirmed previous observations that composite sandwich material is sensitive to thermal influences, which should definitely be taken into account when designing this type of structure.

**Figure 27:**
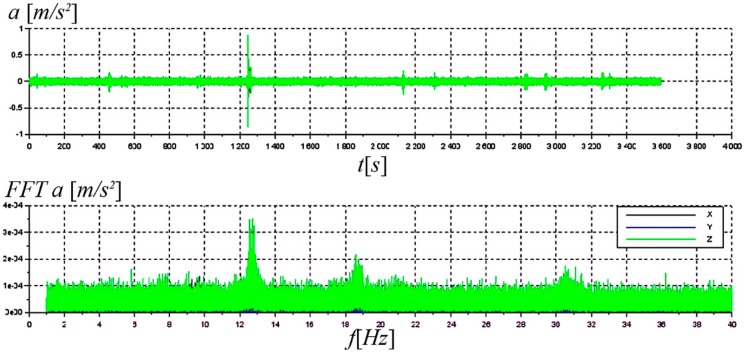
Example data of vibration monitoring with FFT analysis.

While performing the ambient testing, small groups of people only visited the footbridge occasionally. The object was located at the GUT campus beyond the main pedestrian traffic areas and was not used intensively. But the vibration monitoring system was still able to identify some natural frequencies of the structure. The natural frequencies analysis were limited to 40 Hz, due to numerical analysis results (see reference [34]), which showed that the basic structural characteristics fall within this range. The Fast Fourier Transform was obtained automatically with its own system continuously analyzing the recorded signal. In Figure 27 the exemplary measurement data with FFT analysis is presented. It may be observed that the values of around 12.5 Hz and 18.4 Hz are noticeable in the frequency domain. They correspond to the values of 3rd and 5th natural frequencies identified during short-term dynamic tests [34]. The values of natural frequencies did not change throughout the entire research period. On this basis, it can be concluded that the structure global stiffness did not change.

More information about the SHM system of the footbridge can be found in reference [49].

## 6. Conclusions

This paper presents application of different sensors, combined in networks and systems for the purpose of SHM, short, and long-term static tests of novel composite sandwich footbridge. They enable comprehensive analysis of strain, displacement, vibrations, and temperature changes of the bridge, which is very important for engineering structures. The diagnostic approach is based on both standard acceptance procedures for bridge structures made from traditional materials, as well as nonstandard types of tests to confirm that the structure will meet the design requirements throughout its entire intended life.

The strains and displacement measured during the short-term static tests indicated that the structure is safely designed and it has a reserve of stiffness. The predicted numerical values of strains are in good agreement with the ones obtained in real tests, while estimated displacements are mostly higher than measured. All three independently realized load tests (May, September, and November 2015) gave positive results.

The results of a long-term static test (which is not typical for bridge structures), revealed that the three month permanent full design load did not cause negative rheological effects. During the test and after load removal neither stiffness decrease, nor inelastic strains were observed. The registered strain and displacement deviations were caused only by the diurnal and seasonal changes of temperature.

The ambient testing was planned to analyze sensitivity of the bridge to temperature changes. The test covered periods of cold and hot seasons. The registered temperature of structure during this test oscillated between −16 °C and + 38 °C. The results clearly show the impact of both diurnal and seasonal temperature changes. The research confirmed previous observations that the sandwich composite material is sensitive to thermal influences, which should definitely be taken into account when designing this type of structure. The vibration monitoring system proved to be a good tool for assessing global structural stiffness. The values of natural frequencies of an unloaded footbridge were stable.

The results of all multiplied measurements show sufficient compliance. Slight discrepancies may be caused by e.g., not using exactly the same location of measure (too many sensors in the same place). According to the authors’ opinions, all installed sensors have shown usability for application in composite structures.

The analyzed footbridge used a typical research structure when performing the grant. Hence, installation of multiple and rich sensor systems had no improper effect on the use and aesthetics of the object. However, during standard service of a footbridge, such a solution would be impossible for realization. From the feasibility point of view, the least intrusive system in strain measurement is the one based on optic fibers. This system is characterized by a small size of sensors and possibility of their series circuit connection. It even allows us to immerse sensors and most of fiber cables inside a superstructure. The displacement measurement during service of a footbridge may by realized periodically by means of precision leveling or continuously by inclinometers and data post-processing as it is shown in reference [50]. The vibration measurement may be done using the prototype sensors presented in this paper. Even a single six-channels prototype sensor (six orthogonal accelerations, six orthogonal rotational speeds) allows us to determine the basic dynamic characteristics of a structure.

The SHM system allows us to monitor the long-term performance of a loaded as well as an unloaded structure. The SHM used in this case confirmed the design assumptions about footbridge behavior. As a consequence, all groups of sensors could be used to positively assess the exploitation safety of a footbridge. Additionally, knowledge about material behavior could be developed in the future by using a system like the one presented in reference [51].

## Figures and Tables

**Table 1:** Fiber reinforced plastic (FRP) lamina elastic and strength properties.

Elastic/Strength Property	Value
longitudinal elastic modulus E1 [GPa]	23.4
transverse elastic modulus E2 [GPa]	23.4
in-plane shear modulus G12 [GPa]	3.52
transverse shear moduli G13 = G23 [GPa]	2.3
major Poisson’s ratio v12 [-]	0.153
longitudinal strength in tension Xt [MPa]	449
longitudinal strength in compression Xc [MPa]	336
transverse strength in tension Yt [MPa]	449
transverse strength in compression Yc [MPa]	336
in-plane shear strength Sl [MPa]	45.2
transverse shear strength St [MPa]	34.7
